# Rewiring and indirect effects underpin modularity reshuffling in a marine food web under environmental shifts

**DOI:** 10.1002/ece3.5641

**Published:** 2019-09-30

**Authors:** Domenico D'Alelio, Bruno Hay Mele, Simone Libralato, Maurizio Ribera d'Alcalà, Ferenc Jordán

**Affiliations:** ^1^ Department of Integrative Marine Ecology Stazione Zoologica Anton Dohrn Naples Italy; ^2^ Oceanography Division Istituto Nazionale di Oceanografia e di Geofisica Sperimentale ‐ OGS Trieste Italy; ^3^ Balaton Limnological Institute and Evolutionary Systems Research Group MTA Centre for Ecological Research Tihany Hungary

**Keywords:** ecological networks, food webs, modularity, plankton, rewiring, roles

## Abstract

Species are characterized by physiological and behavioral plasticity, which is part of their response to environmental shifts. Nonetheless, the collective response of ecological communities to environmental shifts cannot be predicted from the simple sum of individual species responses, since co‐existing species are deeply entangled in interaction networks, such as food webs. For these reasons, the relation between environmental forcing and the structure of food webs is an open problem in ecology. To this respect, one of the main problems in community ecology is defining the role each species plays in shaping community structure, such as by promoting the subdivision of food webs in modules—that is, aggregates composed of species that more frequently interact—which are reported as community stabilizers.

In this study, we investigated the relationship between species roles and network modularity under environmental shifts in a highly resolved food web, that is, a “weighted” ecological network reproducing carbon flows among marine planktonic species. Measuring network properties and estimating weighted modularity, we show that species have distinct roles, which differentially affect modularity and mediate structural modifications, such as modules reconfiguration, induced by environmental shifts.

Specifically, short‐term environmental changes impact the abundance of planktonic primary producers; this affects their consumers’ behavior and cascades into the overall rearrangement of trophic links. Food web re‐adjustments are both direct, through the rewiring of trophic‐interaction networks, and indirect, with the reconfiguration of trophic cascades. Through such “systemic behavior,” that is, the way the food web acts as a whole, defined by the interactions among its parts, the planktonic food web undergoes a substantial rewiring while keeping almost the same global flow to upper trophic levels, and energetic hierarchy is maintained despite environmental shifts. This behavior suggests the potentially high resilience of plankton networks, such as food webs, to dramatic environmental changes, such as those provoked by global change.

## Graphical abstract

We show, for the first time, the fine‐scale dynamics of structural re‐adjustment in a complex food web, under a regime of changing resources. Our observation is novel, since we investigated directed and weighted modularity in an ecological network, which provides a much sounder view of food web architecture, and therefore functioning; our results are exciting, since food web re‐adjustments shown herein are analogous to those “systemic behaviors” described for other complex systems, such as human brain networks, but an analogous behaviour was never shown in ecological studies with such detail; ultimately, our paper is of general interest in ecology, since it demonstrates that food webs compartmentalization overcomes physical barriers, because species can migrate, and is mainly driven by the aggregation of trophic pathways, more than species co‐occurrence.

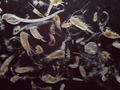

## 1 INTRODUCTION

Individual species are characterized by physiological and behavioral plasticity, which is part of their response to environmental shifts, including those induced by large scale physical and chemical modifications provoked by global change. However, co‐existing species are deeply entangled in interaction networks, such as food webs, in a way that even single‐species behaviors can largely and unpredictably impact the collective response of ecological communities, via indirect effects. Even in light of the dramatic changes our planet is undergoing, evolutionary ecologists—who, by definition, study ecological communities by explicitly considering biological interactions—are increasingly more asked to put species responses within a synthetic, coherent network perspective, in order to predict how changing world will affect the equilibrium of complex ecosystems.

Food webs represent a powerful analytical instrument in the hand of evolutionary ecologists, making it possible to integrate species' biological traits and ecosystem functionality (Thompson et al., 2012). Food webs are “ecological networks” with a fundamental structure, or topology, given by the pattern of species‐species interactions (who is interacting with whom), and a higher‐level structure, or architecture (what is the contribution, or role, of each component to the functioning of the network), which emerges from such a pattern (e.g., Jordán and Scheuring, 2004). The topology of food webs, while constrained by the biological traits of each component, reflects the interplay of local and global structure of interactions. At local level, changes in species and resource abundances regulate the strength of interspecific links, while at the global level network architecture is strongly affected by indirect interactions (Poisot, Stouffer, & Gravel, 2015).

In situ observation, experimental manipulation and computational modeling have suggested that food webs are able to adapt their structure across gradients produced by natural processes, anthropogenic stressors, or both (Tylianakis & Morris, 2017). Thus, a single set of species can display alternative interaction networks based on different standing local conditions (Margalef, 1991; Peacor, Riolo, & Pascual, 2006; Rooney, McCann, & Moore, 2008). This behavior could be explained considering that organisms at lower trophic levels, such as primary producers, play as “oscillators” (due to population's fluctuations) in time and space and tend to occupy fixed positions within specific environments, while higher‐order consumers play as “couplers,” that is, in sorting for available resources they connect different environments. The existence of oscillator and coupler roles allows food webs to display alternative pathways for energy flows, giving rise to “meta‐food webs” able to explore a variety of topologies and architectures during their existence span (Dunne, 2006).

Food web assembly processes often produce an uneven distribution of trophic links among species giving rise to the formation of modules, that is, dense aggregate of links established by species more frequently interacting (e.g., Dormann, Fründ, & Schaefer, 2017; Krause, Frank, Mason, Ulanowicz, & Taylor, 2003; May, 1972). Such a modular organization, promoted by proximate evolutionary determinants—such as coevolving species, diet similarity, and spatiotemporal proximity (e.g., Rezende, Albert, Fortuna, & Bascompte, 2009)—has significant ecological implications; for example, modularity may enhance the persistence of food webs (Stouffer & Bascompte, 2011). In turn, persistence apparently drives different populations to acquire distinct but complementary ecological roles in the course of natural history, as to set a balance between species competition and coexistence (Barabás, Michalska‐Smith, & Allesina, 2017; Kemp, Evans, Augustyn, & Ellis, 2017; Montoya & Solé, 2002; Peacor et al., 2006). Theoretical studies and meta‐analyses showed that highly connected and nested architectures promote stability in mutualistic networks, while modularity is at the base of the stability of antagonistic networks, such as food webs (Thébault & Fontaine, 2010). Thus, studying food webs topology and architecture would allow to analyse the biological drivers behind the network structure and to predict the ecological implications of possible structural changes (Dormann et al., 2017; Ings et al., 2009; Poisot, Canard, Mouillot, Mouquet, & Gravel, 2012). To this respect, one of the main problems in community ecology and evolution is defining the role each species plays in assembling community structure, for example, by promoting modularity reshuffling under sharp environmental modifications.

Within this paper, we aim at investigating the relationship between species roles and network modularity under environmental shifts in a highly resolved food web, such relationship being postulated based on nature observation (Montoya, Yallop, & Memmott, 2015) and modeling exercises (Allesina & Pascual, 2009). Knowledge on networks modifications is sparse and mostly inferred from theoretical models or from undirected, and often unweighted, networks produced by co‐occurrence matrices. In the end, lack of knowledge and scarcely defined networks contribute to keep the relation between environmental forcing and network structure an open problem in ecology.

**Figure 1:**
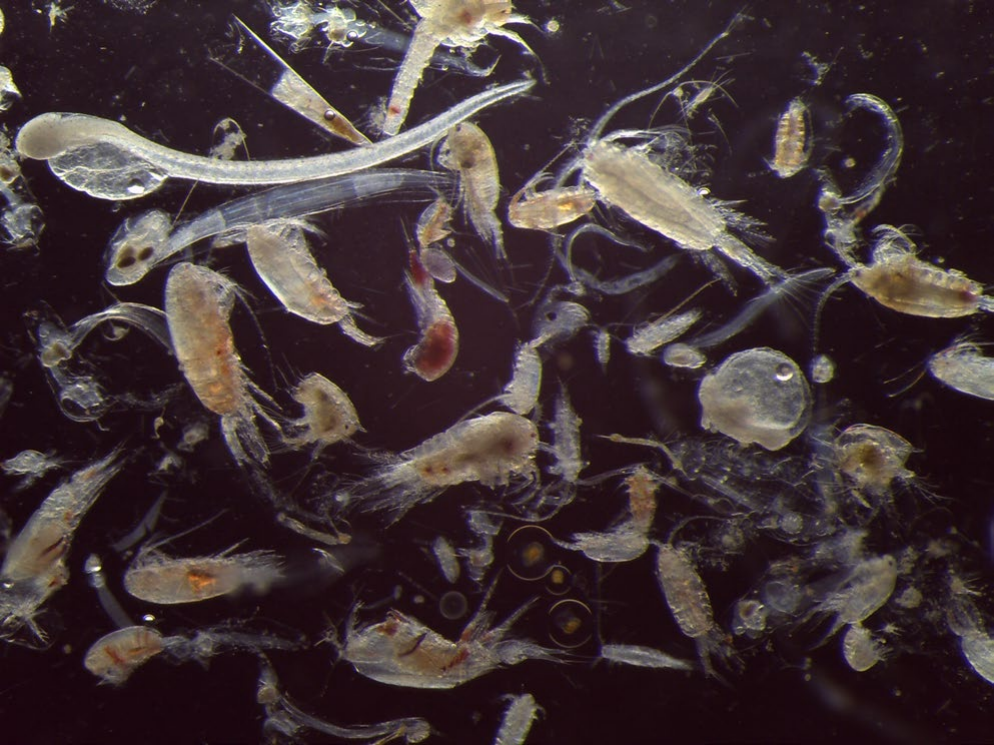
Plankton sample collected in the Gulf of Naples, Italy (courtesy of Iole Di Capua, Stazione Zoologica Anton Dohrn, Naples, Italy)

Our investigation focuses on plankton, a multifaceted group of microscopic organisms living in aquatic environments and including both unicellular and multicellular species (Figure 1). A planktonic food web was computationally defined in two environmental conditions by applying Ecopath network modelling (Christensen & Walters, 2004) to in situ biomass data, as presented previously (D'Alelio, Libralato, Wyatt, & Ribera d'Alcalà, 2016). Measuring network properties and using module detection techniques, we search for: (a) the species roles; (b) the influence of species roles on the structure of the food web; (c) the extent of modularity, and (d) the structural modifications, such as modules reconfiguration, induced by environmental shifts and mediated by changes in species roles. We finally discuss which ecological implications modular changes can have in complex food webs and how relating species roles to food web architecture can support the advancement of ecosystem‐based management in marine ecosystems, in face of the environmental shifts induced by global change.

## 2 MATERIAL AND METHODS

### 2.1 Ecological data

The planktonic food web analyzed in this paper included unicellular organisms (auto‐, hetero‐, and mixo‐trophs) and metazoans sampled from the Long Term Ecological Research “MareChiara” in a coastal marine environment, that is, the Gulf of Naples (GoN in the following; Mediterranean Sea, Italy, LTER‐MC; Ribera d'Alcalà et al., 2004). This web of trophic interactions was derived from a previously published Ecopath model (D'Alelio, Libralato, et al., 2016) in which we published the data required to produce the model outputs further used in the present study.

Ecopath models are particularly suited for studying food webs. Using Ecopath, it is possible to interpolate biomass fluxes across a food web starting from the standing biomasses, physiologies, and diets of the interacting species, ending with an internally coherent and balanced food web model in which link weights are proportional to biomass fluxes throughout the web. Such models provide a synthetic tool for the analysis of fine‐scale properties emerging from the integration of species behavior and their reciprocal relatedness.

The planktonic food web simulated for the GoN was reproduced in two rounds with different inputs referring to distinct environmental conditions, defined for simplicity as “green” and “blue” states (Cianelli et al., 2017). The green state refers to eutrophic conditions due to the impact of fresher coastal waters, richer of inorganic nutrients and phytoplankton biomass, on the surface layers of the inner GoN. Conversely, the blue state refers to the lack of coastal waters impact, which results in lower nutrient input and phytoplankton biomass in the same environment as above. The blue conditions remark those of offshore waters and can be seen as mimicking those predicted by global change studies for coastal environments subject to oligotrophication trends (Agusti, Martinez‐Ayala, Regaudie‐de‐Gioux, & Duarte, 2017; Cloern et al., 2016).

**Figure 2:**
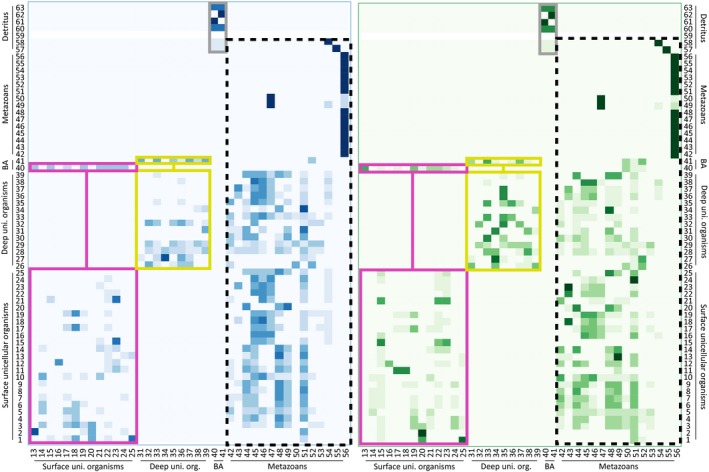
The planktonic meta‐food web from the Gulf of Naples (Italy) described by D'Alelio, Libralato, et al. (2016). In the left and right panels, matrices of carbon flows among predators (in columns) and preys (in rows) for the oligotrophic, or blue, and the eutrophic, or green, states of the food web, respectively. The intensity of squares in the matrix remarks the intensity of the carbon fluxes between predators and preys and, therefore, the weight of the relating trophic link. Pink and yellow boxes include links among unicellular organisms establishing between nodes setting at either surface‐ or deep‐water layers, respectively. Gray boxes include links between bacteria and detritus. Dotted black squares include trophic links between metazoans, which are free to move between surface‐ and deep‐water layers, and unicellular organisms setting at each of these water layers

The plankton model mentioned above simulated the functioning of a food web during the summer season, when the water column resulted as stratified in (a) a surface mixed layer (between 0 and ‐5 m) with higher temperature and lower density; (b) a thermocline, that is, a shallow internal water layer in which temperature underwent a sharp decrease; and (c) a deep‐water layer (below ‐10 m) with lower temperature and higher density. The alternation between green and blue conditions—called “green‐blue swings”—widely affects the biomass budget in the surface mixed layer, while the deep‐water layer remains almost unchanged. According to our model, the plankton food web can quickly respond to green‐blue swings. Indeed, while unicellular organisms were not able to cross the thermocline and resulted physically separated between surface and deep environments, planktonic invertebrates were free to move across the thermocline, thus inducing the reorganization of the food web (Figure 2).

The plankton food web reproduced for the GoN included 63 functional nodes (Table 1) and a total of 718 trophic links. Two distinct predatory matrices visualize the interactions associated with each network in Figure 2. Based on trophic‐link clustering, four potential modules were visually detected in both green and blue predatory matrices (Figure 2; carbon‐flow matrices for this food web are presented in Data S1): two included links between unicellular organisms in either the surface or deep layers; one included links between bacteria and detritus; and one included links between metazoans and all the other nodes. In synthesis, green and blue GoN food webs include the same organisms and share very similar topologies but show considerably different link weights, with the most dramatic changes associated with invertebrates.

**Table 1:** Species present in the plankton food web from the Gulf of Naples

Nodes		Small description		Size (µm)	Blue	Green
					Biomass (mgC/m^2^)	Biomass (mgC/m^2^)
1	Cyanobacteria (s)	Mainly *Synechococcus*	A	1^a^	3.2	4
2	Prochlorophytes (s)	Mainly *Prochlorococcus*	A	1^a^	0.3	0.4
3	Phyto‐nanoflagellates (s)	Several species	A	1.9^a^	22	80.5
4	*Chaetoceros* spp. (s)	Diatom genus	A	2.4^a^	4.2	83.3
5	*Leptocylindrus* spp. (s)	Diatom genus	A	5.8^a^	31.3	317
6	*Skeletonema* spp. (s)	Diatom genus	A	3.1^a^	5.7	47
7	Small diatoms (s)	Several species	A	3.2^a^	4.3	34.1
8	Pennate diatoms (s)	Pennate diatoms	A	3.3^a^	1.2	11.6
9	*Pseudo‐nitzschia* spp. (s)	Diatom genus	A	3^a^	2.3	19.9
10	Centric diatoms (s)	Centric diatoms	A	12^a^	19.7	83.9
11	Coccolithophores (s)	Mainly *Emiliania huxleyi*	A	4.3^a^	3.9	12.3
12	Phyto‐microflagellates (s)	Several species	A	4^a^	3.9	12.9
13	Mixotrophic nanoflagellates (s)	Mainly *Ollicola vangorii*	M	1.5^a^	0.1	0.2
14	Small dinoflagellates (s)	Several species	M	4.5^a^	6.6	23.5
15	Medium dinoflagellates (s)	Several species	M	9^a^	4.1	13.5
16	*Myrionecta rubra* (a)	Ciliate species	M	10^a^	0.6	2
17	*Tontonia* spp. (s)	Oligotrichous ciliate genus	M	40^a^	9.5	35
18	*Laboea* spp. (s)	Oligotrichous ciliate genus	M	22^a^	1.8	6.5
19	*Strombidium* spp. (s)	Oligotrichous ciliate genus	M	38^a^	11.6	34.6
20	HNF (s)	Agglutinated nanoflagellates	H	2.4^a^	0.4	1.3
21	Heterotrophic dinoflagellates (s)	Several species	H	11.1^a^	7.7	48
22	Prostomatids (s)	Agglutinated ciliates	H	26.8^a^	1.7	17.5
23	*Strobilidium* spp. (s)	Ciliate genus	H	26.8^a^	4.3	12.9
24	Tintinnids (s)	Agglutinated ciliates	H	11^a^	0.2	1.7
25	Nanociliates (s)	Agglutinated ciliates	H	8^a^	0.7	2.3
26	Cyanobacteria (d)	Mainly *Synechococcus*	A	1^a^	108.4	155.9
27	Prochlorophytes (d)	Mainly *Prochlorococcus*	A	1^a^	10.8	15.6
28	Phyto‐nanoflagellates (d)	Several species	A	1.9^a^	33.6	48.3
29	Coccolithophorids (d)	Mainly *Emiliania huxleyi*	A	4.3^a^	166.2	239
30	Diatoms (d)	Several species	A	3.2^a^	10.3	14.7
31	Mixotrophic nanoflagellates (d)	Several species	M	1.5^a^	0.1	0.1
32	Small dinoflagellates (d)	Several species	M	4.5^a^	85.5	108.2
33	Medium dinoflagellates (d)	Several species	M	9^a^	52.9	62.3
34	HNF (d)	Agglutinated nanoflagellates	H	2.4^a^	0.1	0.1
35	Heterotrophic dinoflagellates (d)	Several species	H	11.1^a^	34.2	44.6
36	Prostomatids (d)	Agglutinated ciliates	H	26.8^a^	7.3	16.2
37	*Strobilidium* spp. (d)	Ciliate genus	H	26.8^a^	19.1	12
38	Tintinnids (d)	Agglutinated ciliates	H	11.4^a^	1	1.6
39	Nanociliates (d)	Agglutinated ciliates	H	8^a^	3	2.1
40	Heterotrophic bacteria (s)	–	H	0.5^a^	32.7	108.5
41	Heterotrophic bacteria (d)	–	H	0.5^a^	373.5	397.3
42	*Penilia avirostris* (a)	Cladoceran species	H	800^b^	96.1	100.8
43	Cladocerans (a)	*Evadne* & *Pseudevadne* spp.	H	900^b^	33.8	65.7
44	*Paracalanus parvus* (a)	Calanoid copepod species (adults)	H	850^b^	25.5	26.8
45	*Acartia clausii* (a)	Calanoid copepod species (adults)	H	1,150^b^	7.5	22
46	*Temora stylifera* (a)	Calanoid copepod species (adults)	H	1,000^b^	39.1	37
47	*Centropages typicus* (a)	Calanoid copepod species (adults)	H	1,000^b^	12.2	24.6
48	Other calanoids (a)	Agllutinated genera (adults)	H	1,050^b^	8.7	7.7
49	Juvenile calanoids (a)	Juveniles of calanoid copepod	H	450^b^	14.6	21.2
50	Appendicularia (a)	Agglutinated species	H	3,000^b^	36.1	39.8
51	Doliolids (a)	Agglutinated species	H	1,500^b^	2	3.7
52	Salps (a)	Agglutinated species	H	10,000^b^	16.2	30.8
53	Meroplankton (a)	Agglutinated larvae	H	250^b^	3.5	4.7
54	*Oithona* spp. (a)	Cyclopoid copepod genus	H	675^b^	1.4	1.3
55	Detritivora (a)	Cyclopoid copepod genera	H	650^b^	7.4	5.2
56	Carnivora (a)	Mainly chaetognats	H	28,000^b^	276.3	295.5
57	Appendicularia houses (a)	–	D	3,000^b^	113.8	489.9
58	Small fecal pellets (a)	Feces of small animals	D	<200^b^	81.5	396.5
59	Salp fecal pellets (a)	Fecal pellets of salps	D	>200^b^	3.8	7.3
60	Carnivores F.P. (a)	Fecal pellets of carnivores	D	>200^b^	0.6	1.2
61	DOC (s)	Dissolved Organic Carbon	D	<0.2^b^	16.6	102.9
62	DOC (d)	Dissolved Organic Carbon	D	<0.2^b^	58.3	81.9
63	Generic particulate detritus (a)	Amorphous particulate detritus	D	<200^b^	4,486.8	2,629.7

Notes

(s) Living in the surface‐water layer. (d) Living in the deeper water layer. (a) Living all over the water column.

Abbreviations: A, autotrophic; D, detritus; H, heterotrophic; M, mixotrophic.

a Equivalent Sphere Diameter (average).

b Length (average).

### 2.2 Network roles

We inspected the roles of different nodes in the green and blue food webs by employing indices developed for the analysis of weighted networks (see Scotti, Podani, & Jordán, 2007). By ranking nodes based on network metrics, we assessed species roles and the switching of these latter between the two webs. Analytically, we used a combination of the following network‐analyses metrics: weighted indirect net effect (*INE*); weighted centrality (*WI*); and weighted overlap (*WO*).

*INE*, by definition, represents the overall indirect weighted impact that a group has on the entire network, and it has very similar properties to those of the overall effect used to identify keystone species in food webs (Libralato, Christensen, & Pauly, 2006). Further details on the mathematical formulation for the *INE* index can be found in Data S3.

The *WI* index expresses the central position of nodes in the food web. In turn, the *WO* index can be considered as a measure of trophic niche overlap, and a low‐*WO* rank indicates a high trophic uniqueness. While centrality (*WI*) suggests that richly connected species can be important, overlap (*WO*) reflects an early definition of keystone species (single‐species functional groups, see Bond, 1994) and suggests that species that cannot be easily replaced by others are also of crucial importance (for similar methods, see Luczkovich, Borgatti, Johnson, & Everett, 2003; Shannon & Cury, 2004). In calculating these indices, we assumed a network with undirected links where trophic effects could spread in any directions without bias. The reason for this is that we were interested in interaction webs, in the broadest sense, not only bottom‐up trophic flows. Indeed, indirect effects can spread in both bottom‐up and top‐down directions across trophic links.

The metrics *WI* and *WO* were derived from the methods of Godfray and colleagues (Morris, Lewis, & Godfray, 2004; Muller, Adriaanse, Belshaw, & Godfray, 1999; Müller & Godfray, 1999; Rott & Godfray, 2000). For the calculations of *WI* (see also Jordán, Liu, & Veen, 2003), we considered *n* = 3 (maximum three steps for indirect effects), and we used the CosbiLab Graph software for the calculations (Valentini & Jordán, 2010). As for *WO* (see also Jordán, 2009), we used the CosbiLab Graph software for the calculations (Valentini & Jordán, 2010). Further details can be found in Data S3.

### 2.3 Modularity changes

The matrices of trophic links for the green and blue networks were plotted using R‐generated heatmaps (R Development Core Team, 2008) in which color scaling was representative of variabilities in links' weights.

We used the Infomap algorithm (Rosvall, Axelsson, & Bergstrom, 2009) to cluster nodes into nonoverlapping modules. Infomap is a diffusion‐based technique that considers a community as a group of nodes where a random walker is more likely to be trapped in; the Infomap algorithm chooses the best network partitioning by optimizing the random walk description length through the comparison of compressibility of different random walks (Rosvall & Bergstrom, 2008). We applied Infomap to the green and blue food webs separately and instructed the algorithm to take into account node weight (i.e., biomass), to include self‐links (i.e., cannibalism), to assume directed link, and to consider the link weights (i.e., the carbon flow) for guiding the random walker.

We chose Infomap because of its consistency (Lancichinetti & Fortunato, 2009) and performance (Fortunato & Hric, 2016) and because common detection methods via modularity maximization do not consider direction and weight. Furthermore, the concept behind the algorithm, that is, a random walk guided through nodes by an information flux, is biologically meaningful, as it can be assimilated to the carbon flowing through the trophic network. A module in our trophic network can be regarded as a cluster of nodes (within food web compartmentalization) among which carbon flows smoothly, and it is thus equivalent to a single trophic compartment (meta‐node).

The module membership vector produced by Infomap was then used to inform the network visualization in Gephi (Bastian, Heymann, & Jacomy, 2009) using the Fruchterman–Reingold Algorithm, a force‐directed layout algorithm (Fruchterman & Reingold, 1991). Nodes size was set as proportional to “weighted degree,” that is, a measure of node's interconnection based on the weight of links to node's neighbors. We also built an alluvial diagram to depict how the different nodes redistribute among the modules as the network shifts from the blue configuration to the green one; we represented these modules as rectangles and groups of nodes shifting between modules as stream fields. The thickness of the field was set as proportional to the group contribution to the module outflow.

### 2.4 Direct–indirect effects

Based on the *WI* index, not only key species but also key interactions were identified. In a network of *n* = 62 nodes (since node #59 was isolated), such as the one investigated herein, *n*(*n*−1) = 3,782 directed effects were realized between species i and j. Out of these, 1,248 ij interactions were direct (included in a predation matrix and shown in the food web), and the rest were indirect. The *WI* index did not consider the direction of links in the food web, so the spread of effects was calculated in all directions.

After ranking the strength of these interactions, we selected the ones that were stronger than an arbitrary limit of 0.001 and assembled them into specific networks using the yEd graph editor (yFiles software; Wiese, Eiglsperger, & Kaufmann, 2004) to display the regulative “network cores” (sensu Daily, Ehrlich, & Haddad, 1993; Ortiz et al., 2013). While most interactions were similar between nodes i and j (ij and ji were both strong or both weak), some pairs of nodes were in an asymmetric relationship: This was indicated by different dimensions of arrow tips in the yEd networks. Further details can be found in Data S3.

## 3 RESULTS

**Figure 3:**
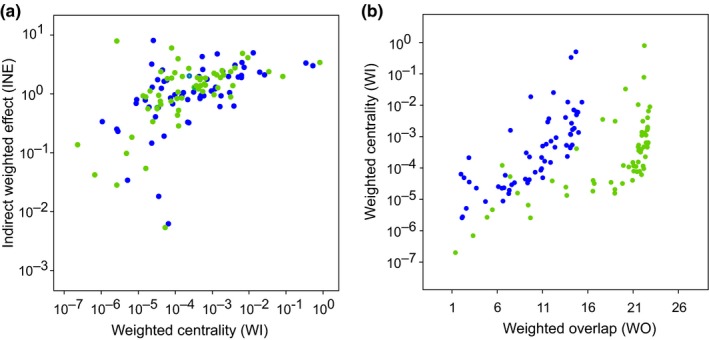
Network roles analyses for the planktonic food web in the Gulf of Naples (Italy) at oligotrophic, or blue, and eutrophic, or green, states. In both (a) and (b) graphs, green and blue dots refer to web nodes at those different environmental conditions. (a) The relationship between indirect weighted effect (*INE*) and weighted centrality (*WI*). (b) The relationship between weighted centrality and overlap (*WI* and *WO*, respectively)

Most nodes in the planktonic food web from the GoN modified their positional importance between the blue and green states (Figure 3). Among network metrics investigated herein, *WI* and *INE* displayed definite covariance patterns at both states (Figure 3a), suggesting that changes in nodes centrality (*WI*) were able to affect also the impact that nodes exerted over the whole food web (*INE*). The relation between *WI* and *WO* was nonlinear and seemingly hyperbolic: for higher values of *WO*—and, therefore, decreasing uniqueness of nodes—*WI* strongly increased. In synthesis, we observed that *WO* was larger in green state and changes with *WO* were discontinuous. Larger *WO* meant multiple trophic solutions, while transition between large resources (green) and low resources (blue) states reduced the number of solutions, that is, by inducing trophic specialization.

**Figure 4:**
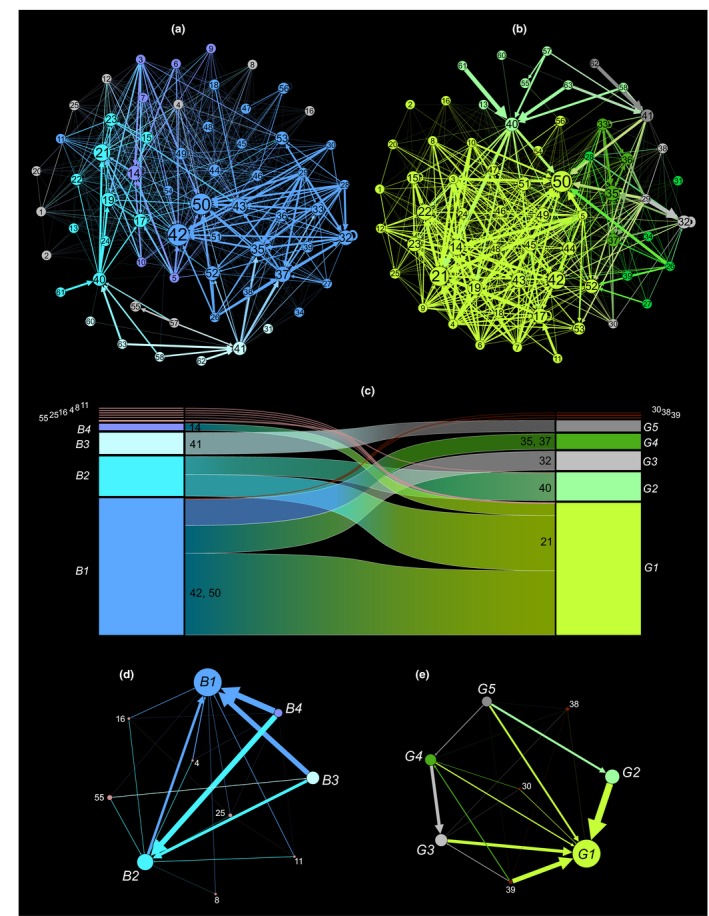
Modularity reshuffling in the planktonic food web from the Gulf of Naples (Italy) between oligotrophic, or blue, and eutrophic, or green, states. (a, b) Weighted networks derived for the planktonic food web at blue and green states, respectively, produced by the Gephi software (Bastian et al., 2009) using the Fruchterman–Reingold, force‐directed layout algorithm (Fruchterman & Reingold, 1991). Network nodes are different species or species groups of the food web, as indicated in Table 1; nodes' dimension is proportional to their weighted degree as estimated by Gephi; links' weight is proportional to the dimension of carbon flows among web nodes; and nodes colors remark their association to different modules, as based on weighted and directed modularity analyses (see M&M's). (c) Alluvial diagram depicting how the different nodes redistribute among the modules as the network shifts between blue and green configurations; colors are alike those in (a, b) and indicate the main network modules, which are represented as rectangles whose dimension is proportional to the fraction of carbon flows within each module. Groups of nodes shifting between modules at blue‐green transitions are represented as stream fields, whose thickness is proportional to the group's contribution to the module outflow; light and dark red streams indicate the translation of nodes belonging to recessive modules made of few or a single node. (d, e) Aggregated net fluxes among modules in simplified networks for the blue and green networks, respectively; colors are as in (a–c), nodes are modules, nodes' size is proportional to the fraction of carbon flow within each module, and links' weight is proportional to the dimension of carbon flow among modules

Figure 4 shows the two weighted networks derived for the planktonic food web at blue and green states, their weighted modularity, the transitions of some nodes between modules in the course of green‐blue swings, and the aggregated net fluxes among modules. The blue and green networks were different in terms of: (a) node‐ranking (as weighted degree, mirrored by nodes' size in Figure 4a,b); (b) pattern of link‐clustering; and (c) number of the main modules, which were four and five in blue and green webs, respectively (coded as B1‐4 and G1‐5 in Figure 4c), when excluding minor modules constituted by few or even single, outlying nodes. Moreover, both main modules G1 and B1 included several unicellular nodes from either surface‐ or deep‐water layers, respectively, and the weighted and directed modularity did not respect physical compartmentalization (compare with Figure 2).

Modularity in blue and green states had some common general features: (a) both networks showed one dominant module, either B1 or G1, which aggregated almost 64% of the overall biomass fluxes; (b) these modules were dominated by node #50, the pelagic tunicates Appendicularia; and (c) together with other animals (e.g., #42‐43, 52), node #50 was present in the dominant (energy‐wise) module of each system state. Modularity reshuffling at blue‐green shifts was evident from the alluvial analysis. From blue to green states (Figure 4c): (a) nodes #32, 35, and 37, that is, unicellular consumers setting at the deeper water layer, left the main module (B1) and formed two secondary modules (G3‐4); (b) nodes #21 and 40, that is, heterotrophic dinoflagellates and bacteria, both setting in the surface‐water layer, left the second‐in‐rank blue module B2, and whereas #21 entered the main green module G1, #40 produced a new secondary module, G2.

Meta networks were built by aggregating net biomass fluxes among modules only (Figure 4d,e): therein, the blue modular web was almost “bipolar,” that is, it included two main providers (B3, B4), and two main utilizers (B1 and B2). On the other hand, the green modular web was “unipolar,” with G1 attracting most biomass fluxes from five providers (G2‐5). By comparing complete and simplified graphs (panels a‐b and d‐e in Figure 4), it is worth noticing that the blue network is more linear than the green one, which appears as relatively intricate. This aspect remarks the presence of multiple trophic pathways at green state, as suggested by the higher *WO* values in respect to the blue state (see Figure 3b).

**Figure 5:**
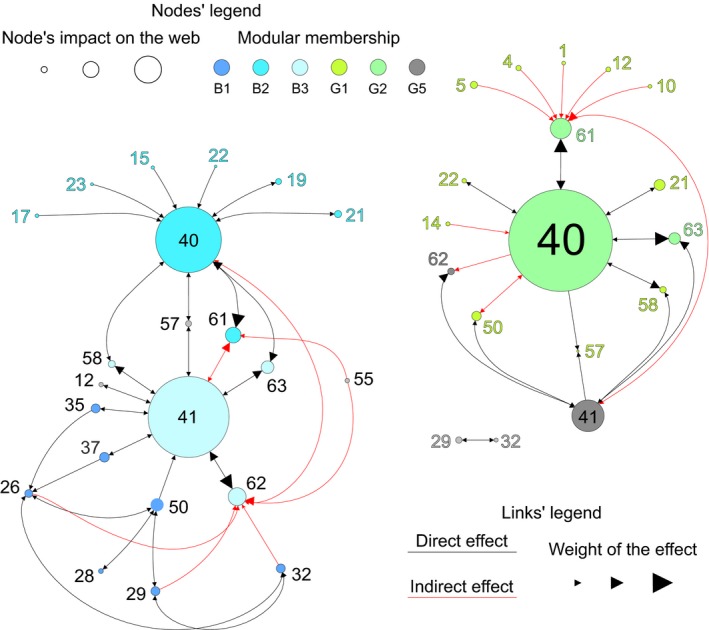
Direct and indirect effects in the core parts of the networks of the plankton food web in the Gulf of Naples (Italy). Nodes–nodes effects are based on the *WI* index. Plankton webs refer to oligotrophic, or blue, and eutrophic, or green, states (a, b, respectively). Core parts were defined following previous authors (Daily et al., 1993; Ortiz et al., 2013). Nodes are species or groups of species as indicated in Table 1; different colors (as in Figure 3) indicate the membership of nodes to different modules; nodes' size is proportional to the absolute impact of a node on the web; black and red links are direct and indirect effects, respectively. The yEd graph editor (yFiles software; Wiese et al., 2004) was employed to visualize these networks

Figure 5 shows the two core part of the networks derived from the strongest interactions that were detected in the blue and green states, respectively, based on the *WI* index. The core parts are related to the heterotrophic/detrital components of the networks and show distinct structure of direct and indirect interactions. The strongest interactions in the blue core were from deep bacteria to deep DOC (#41 and #63) and from surface bacteria to surface DOC (#40 and #61), and both were direct (trophic); deep DOC (# 62) was the strongest indirect interactor, involved in 5 out of 9 total indirect effects. In the green core, the strongest direct (trophic) interaction was from surface bacteria and surface DOC (#40 and #61, respectively); the latter was also the strongest indirect interactor, being involved in 7 out of 11 total indirect effects. In both cores, indirect effects involved nodes belonging to different modules.

Data matrices for *INE*, *WI,* and *WO* are presented in Data S2.

## 4 DISCUSSION

We investigated the relationship between species roles and network modularity under sharp environmental shifts in a highly resolved plankton food web simulated by an Ecopath model previously published (D'Alelio, Libralato, et al., 2016). By measuring network properties, we revealed that plankton species have distinct roles, which differentially mediate structural modifications, such as modules reconfiguration, induced by environmental modification. Specifically, short‐term environmental changes impact the abundance of planktonic primary producers: This affects consumers' behavior and cascades into the overall rearrangement of trophic links. Food web re‐adjustments are both direct, through the rewiring of trophic‐interaction networks, and indirect, with the reconfiguration of trophic cascades, which is particularly relevant in coastal systems, such as the GoN (D'Alelio, Libralato, et al., 2016; D'Alelio et al., 2015). Through such “systemic behavior,” the planktonic food web undergoes a substantial rewiring while keeping almost the same global flow to upper trophic levels, since energetic hierarchy is maintained despite environmental shifts. This finding suggests the potentially high resilience of planktonic communities to dramatic environmental changes, such as the oligotrophication potentially induced by climate change impacts on coastal environments (Agusti et al., 2017; Cloern et al., 2016).

Planktonic environments are influenced by water transport and mixing. At the same time, planktonic communities are deeply affected by the water stratification entailing habitat fragmentation (e.g., Cianelli et al., 2017). Short‐term environmental changes impact the abundance of planktonic primary producers, ultimately resulting in the rearrangement of trophic links involving their consumers. Food web adjustments are both direct, through the rewiring of trophic‐interaction networks, and indirect, with the reconfiguration of trophic cascades. Such topological changes may propagate to higher levels of organization (i.e., the network architecture), contributing to alter modules' composition. Ecological networks are already known to change in time and space (Poisot et al., 2015; Trøjelsgaard & Olesen, 2016), and marine food web structures are known to vary along environmental gradients (Kortsch et al., 2019). Nonetheless, modularity reshuffling is not generally assumed (e.g., Caputi et al., 2019; Guidi et al., 2016) and seldom reported in ecology (Pilosof, Porter, Pascual, & Kéfi, 2017), although it is a well‐known behavior of complex systems. In human brain networks, for instance, learning can be promoted by the flexibility of synaptic links and selection toward optimal neural pathways gained by means of additive steps (Bassett et al., 2011).

Modularity reshuffling in planktonic food webs is realized via what we can call *systemic behavior*. This is the translations of some biological nodes—in general, those playing less central network roles—between different modules; in turn, some other nodes—in general, those playing more central roles—maintain their reciprocal positions, as exemplified by alluvial graph in Figure 4c and core networks in Figure 5. Under oligotrophic and eutrophic conditions (i.e., blue and green states, respectively), the GoN plankton food webs showed variation in the extent of flows, but kept similar structure of direct and indirect effects through internal adjustments. As a result, the planktonic food web underwent a substantial rewiring while maintaining almost the same global flow to upper trophic levels, since energetic hierarchy was maintained despite environmental variability, as suggested for other ecological systems (Kemp et al., 2017). To this latter respect, the more energetic modules, that is, G1 and B1, at the green and blue states of the planktonic food web included mostly invertebrates (see Figure 4), which compose the basic diet of small pelagic fish standing at the top of that food web (see also D'Alelio, Montresor, et al., 2016).

Our results indicate that indirect effects further reinforce the maintenance of this hierarchy by setting negative feedbacks. This observation suggests the existence of a strong, though poorly explored in nature, relationship between species roles and the architecture of food webs. In the following sections, we will discuss in detail the fine‐scale mechanisms at the base of structural reorganization of planktonic food webs, which are pursued by the diversity of species roles, as network positional importance and indirect impact over the web. Moreover, we discuss how our results may translate in a more effective assessment of food webs state in pelagic ecosystems.

### 4.1 Food web rewiring, indirect effects and modularity reshuffling

**Figure 6:**
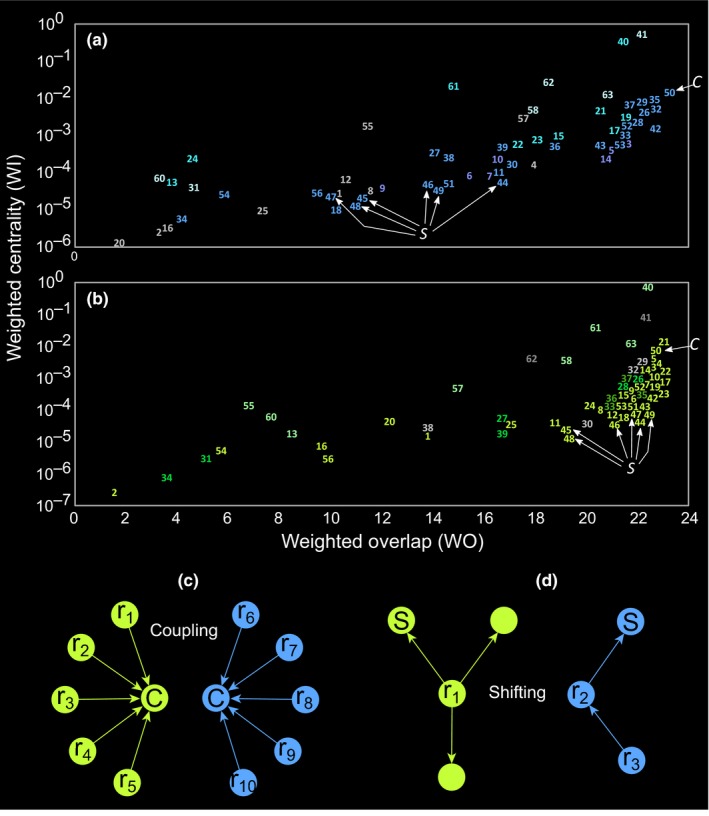
Species roles variability in the planktonic food web from the Gulf of Naples (Italy) at transitions between oligotrophic or blue and eutrophic or green states. (a, b) Relationships between weighted overlap (*WO*) and weighted centrality (*WI*) for web nodes at blue and green states, respectively; numbers are nodes id (see Table 1) and their position indicate nodes position in the x‐y plot; colors are as in Figures 3 and 4; and white arrows indicate “C” and “S” nodes, that is, “couplers” and “shifters” animals, respectively, with the first not modifying their network position and the second modifying their (niche) overlap (*WO*) at blue‐green shifts. (c) Coupling behavior in a consumer node such as Appendicularia (#50 in a, b; herein, this node is coded as “C”). When the system shifts between green and blue states, a coupler, that is, a highly generalist consumer, undergoes strong rewiring, from resources nodes “r_1‐5_” to resources nodes “r_6‐10,_” and it remains at the center of fluxes—as a consequence, its positional importance does not change, as well as its niche overlap. (d) Shifting behavior in a consumer node such as calanoid copepods (#44‐49 in A‐B; herein, this node is coded as “S”). When the system shifts between green and blue states, a shifter, that is, a highly selective consumer, shifts between distinct trophic pathways—as a consequence, its positional importance largely changes, as well as its niche overlap, which is higher at green than blue states. Notice that nodes without codes are other consumers competing with the shifter node, while r_1‐3_ nodes are resources nodes

In the planktonic community investigated herein, most higher‐level consumers (#42‐56) occupy the more energetic module in both blue and green states, since they aggregate where much food is available (Figure 4). Notably the nodes showing the highest centrality (expressed by the *WI* index) at both eutrophic and oligotrophic states have a higher impact on the web (see the position of nodes #42‐56 in Figure 6). In addition, the ability of species to change their modular membership between different trophic states, which is remarked by their relatively high overlap (*WO* index), not only supports the hypothesis of plankton animals as flexible in terms of trophic preferences, but also invokes for their systemic importance, that is, concentrating different fluxes at different system states, modifying the composition of modules concerning aggregated links, etc. In more specific terms, behavioral plasticity at species level—that is, different animals show a breadth of trophic strategies based on the characteristics of the actual “food environment”—stands at the base of a community plasticity, which manifests trough modularity reshuffling in the plankton food web: This systemic behavior allows quick responses to sharp environmental shifts by considerably expanding the “Reaction gamma”—that is, the range of alternative food web and ecological networks architecture generated by different environmental states.

Among higher‐level consumers, Appendicularia (node #50) show high trophic niche overlap (*WO*)—the highest among all the species—and higher centrality (*WI*) qualify them as energy hub regardless of the system state. These generalist filter‐feeding invertebrates can undergo strong rewiring between states, persist in their centrality role in the network of fluxes by interacting with nodes belonging to different modules that permit to switch (Figure 6). A similar relationship between trophic performances and system organization is found in forest soils: Therein, generalist invertebrates show a highly redundant network position at habitat edges and this allows extensive rewiring of interaction networks based on a nonrandom, apparently adaptive, dynamics (Peralta, Frost, Didham, Rand, & Tylianakis, 2017). In the course of green‐blue transitions in GoN plankton community (D'Alelio, Libralato, et al., 2016), Appendicularia can feed in the main energetic module of the food web, including either surface or deep unicellular nodes, based on their relative availability: To this respect, Appendicularia may behave as “couplers” sensu Rooney et al. (2008) (Figure 6c). This hypothesis is in line with field observations: Like zooplankton of similar size, appendicularians perform vertical migrations throughout the water column following higher food concentrations (Ursella, Cardin, Batistić, Garić, & Gačić, 2018) and this strategy can be at the base of the explosive demographic increases observed for these and other pelagic tunicates (e.g., Conley, Lombard, & Sutherland, 2018).

At the other end of the spectrum, calanoid copepods (#44‐49) undergo considerable changes in trophic niche overlap (*WO*; Figure 6a,b), that is, they are in a unique network position in the blue regime. Even though calanoids are not energy hubs of the system we investigated, their role is relevant: By being more trophically specialized at blue system states, their presence guarantees robust planktonic food webs at oligotrophic conditions. While Appendicularia regulate the extension of the main energetic module, calanoids keep the internal cohesion of this module by modifying their trophic behavior at blue‐green transitions: To this respect, they play as “shifters” sensu Margalef (1991) (Figure 6d). Copepods are reported as resilient to changing conditions in different marine systems (Mazzocchi, Dubroca, García‐Comas, Capua, & Ribera d'Alcalà, 2012; Paffenhöfer, Sherr, & Sherr, 2007) and have been considered as energy gates, linking different trophic levels and switching between alternative pathways (Stibor et al., 2004). In the GoN, calanoids are reported to guarantee an effective flow of matter toward small pelagic fish by changing dietary preferences based on resource availability (D'Alelio, Montresor, et al., 2016).

Beside biological characteristics and consequent modulation of the trophic activities of some key organisms, the structure of food webs is regulated by indirect modifiers, such as indirect effects or trophic cascades (Barabás et al., 2017; Poisot et al., 2015; Schmitz, Krivan, & Ovadia, 2004). In our investigation system, planktonic primary producers in the surface waters (i.e., they are resource nodes in module G1, Figure 4) induce a substantial effect on surface DOC (module G2, Figure 5). It is worth noticing that DOC, that is, the primary food of surface bacteria (#40, module G2, Figures 4 and 5), is released in large quantities by primary producers, mainly in eutrophic conditions (Wear et al., 2015). Also, as a consequence of the limited compartmentalization of surface‐ and deep‐water habitats, bacterial nodes indirectly influence each other by affecting each other's food, that is, DOC. As a consequence, indirect effects crossing borders between modules can keep different modules in connection while maintaining their energetic compartmentalization. As for our elaborations, indirect effects appear as affecting the opening and the release of the network structure at system state transitions. For instance, the multidirectional indirect effects exerted by module #1 on module #2 provoke a tighter clustering among these modules in the green than in the blue networks (Figure 5).

### 4.2 New indicators for food webs state?

The systems approach allow dealing with ecological complexity in a simplified fashion by estimating the relative importance of different co‐existing organisms. This approach offers a rigorous and unbiased evaluation on potential key species and interactions in the face of environmental changes (Jordán, 2009). Testing new network metrics suitable to derive ecological indicators via complex systems analyses is of primary importance in marine ecology, in light of the increasing availability of data that flanks the rising of the so‐called meta‐omics era (D'Alelio et al., 2019). Considering the present study, it is worth noting that weighted overlap (*WO*) reveals to be a good indicator of environmental changes: It quantifies the uniqueness versus the redundancy of the network neighborhood of nodes, and it is also of evolutionary relevance, being a measure of trophic niches. Finally, it helps categorize organisms based on their network importance.

For instance, Appendicularia are essential hubs in plankton networks and can be successful players in the future oceans (Bouquet et al., 2018; Winder et al., 2017). Nonetheless, in our analyses, they did not show significant positional differences between the two states of the system investigated herein (Figure 6a,b). They can be key players with roles changing in time (sensu Banerjee, Scharler, Fath, & Ray, 2017) but not appropriate indicators of system shifts. In fact, surface heterotrophic nanoflagellates (#20) showed the most substantial positional change in the two conditions investigated herein (Figure 6). We do not know whether they are active drivers of systemic changes or passive followers of these, but they are better systemic indicators than Appendicularia. In the same way, planktonic nanoflagellates showed in our study a high adaptive potential to changing environmental conditions also coherent with other works (Moustaka‐Gouni, Kormas, Scotti, Vardaka, & Sommer, 2016). Planktonic nanoflagellates are also suspected of mixotrophy, that is, a metabolism shifting between auto‐ and phagotrophy (Stoecker, Hansen, Caron, & Mitra, 2017), a successful strategy in oligotrophic oceans (Hartmann et al., 2012) that give scope for adaptation.

The analysis of interaction strengths performed herein showed that some indirect effects were significantly stronger than many direct/trophic interactions. These effects were similar but not merely the same as the largest carbon flows in the system, and the web nodes involved in these important effects formed the regulative core of the community (Figure 5). Considering indirect interactions is therefore crucial for the better understanding of the ecosystem functioning, beyond their potential for quantifying cascading effects and envisioning possible secondary extinctions. Indirect interactions, in fact, also regulate the structural modifications needed for assuring functionality of the food webs in changed conditions by limiting the rewiring and reshuffling and keeping the main hierarchical structure of the system.

Ecologists often estimate ecosystem robustness with regard to physical compartmentalization, which would limit the spreading of perturbations (Grilli, Rogers, & Allesina, 2016). Our work demonstrates that food webs compartmentalization can overcome physical barriers, because species migrate in search for food, and module assembly is mainly driven by the aggregation of trophic pathways, more than species co‐occurrence. Therefore, when putting links' clustering within a trophic/energetic context—that is, by analyzing weighted modularity and not the simpler nodes co‐occurrence—physical compartmentalization decreases in importance and becomes only a component of modular units defined at a higher systemic level. In addition, the reshuffling of “energetic” modularity allows maintaining a hierarchical structure despite the different interaction networks that a complex food web, like the planktonic one investigated herein, can display at different conditions. Such an energetic compartmentalization could be an important determinant of ecosystem stability and should be investigated further in food webs. On the contrary, co‐occurrence networks provide a distorted view of the architecture, and therefore, functioning, of the web.

### 4.3 Ecological determinants of plankton networks

Our work calls for the definition of a novel set of indicators based on network metrics suitable for ecosystem‐based management by providing a synthetic view of ecosystem changes. Structural changes in food webs are increasingly reported in consequence of anthropogenic environmental modifications (Tylianakis & Morris, 2017). To this respect, planktonic food webs reveal to be promising study system for investigating mechanisms behind those changes. Plankton communities are characterized by a substantial biological, trophic, complexity (D'Alelio, 2017); such a complexity cascades into convoluted interaction networks, whose characteristics can change in both time and space (D'Alelio, Libralato, et al., 2016; D'Alelio et al., 2015; Guidi et al., 2016; Lima‐Mendez et al., 2015). In principle, studying planktonic food webs have the advantage of analyzing fast processes (compared with higher trophic levels) but also the disadvantage of being poorly known and difficult to observe—even though omics techniques can provide deeper biological information of biological interactions (D'Alelio et al., 2019). Therefore, short time series can be used to understand effects of processes on community structure, whereas system analyses can provide early warning signals.

Most works on plankton systems often use a simplified scheme based on plankton functional types (PFT; Le Quéré et al., 2005) and thus a small number of already compartmentalized functions. Yet, studying how trophic diversity of plankton organizes in time and space has the advantage of exploring mechanisms behind processes that are overlooked by classical plankton models. For instance, the changing of species roles at green‐blue transitions allows nonlinear re‐adjustments in the plankton food web. We show that, from green to blue states, despite a seven fold decrease in phytoplankton biomass (i.e., the resource at the lowest food web level), planktonic animals keep on concentrating the available biomass by taking it from intermediate levels of the web, and this allows to stabilize the energetic hierarchy of the food web. For instance, from green to blue states the animals herein defined as “switchers” increased their predation on protozoa (#13–25), which stand at the intermediate level of the food web, from ~33% to ~41% of their total daily consumption and this allowed them to compensate the possible negative effects emerging from the phytoplankton decrease. Lacking a well‐resolved food web scheme, the PTF does not include such kind of nonlinear responses and it is therefore weakly suited to reproduce the functioning of planktonic systems.

Results presented herein could be representative for processes occurring in other complex ecological systems under perturbation: The effects are detected using synthetic metrics and descriptors (see Link et al., 2015) but often processes behind are difficult to disentangle for the long delays of higher trophic levels population dynamics, which can be also largely impacted by indirect effects (e.g., Agnetta et al., 2019). Our work calls for further efforts in increasing the resolution when investigating the bottom and the middle of pelagic food webs, that is, where plankton stand. To this respect, system approaches must be applied to evaluate how much sensitive to changes the marine food webs are, in the face of global change. If extended to other relevant ecological systems, such kind of approach could significantly aid to understand how a changing world will affect the properties of complex ecosystems—such as stability, persistence, resilience, and matter flow—therefore allowing evolutionary ecologist to better predict how these properties will shift and what the implications are for the wider ecosystem and environment.

## CONFLICT OF INTEREST

None declared.

## AUTHOR CONTRIBUTION

DDA, MRdA, and FJ conceived the study. DDA and FJ collected the data. DDA, FJ, BHM, and SL analyzed the data. DDA wrote the manuscript with substantial input from FJ, BHM, SL, and MRdA.

## Data Availability

Data pertaining to the production of the plankton food web analyzed in this paper are published and commented in D'Alelio, Libralato, et al. (2016); https://doi.org/10.1038/srep21806) and D'Alelio, Montresor, et al. (2016); https://doi.org/10.4081/aiol.2016.5646); the biomass flow matrices analyzed herein are appended in Data S1 of the present article; single values of the network indices presented herein are appended in Data S2.

## References

[ece35641-bib-0001] Agnetta, D. , Badalamenti, F. , Colloca, F. , D'Anna, G. , Di Lorenzo, M. , Fiorentino, F. , … Libralato, S. (2019). Benthic‐pelagic coupling mediates interactions in Mediterranean mixed fisheries: An ecosystem modeling approach. PLoS ONE, 14(1), e0210659 10.1371/journal.pone.0210659 30645620 PMC6333361

[ece35641-bib-0002] Agusti, S. , Martinez‐Ayala, J. , Regaudie‐de‐Gioux, A. , & Duarte, C. M. (2017). Oligotrophication and metabolic slowing‐down of a NW Mediterranean coastal ecosystem. Frontiers in Marine Science, 4, 432 10.3389/fmars.2017.00432

[ece35641-bib-0003] Allesina, S. , & Pascual, M. (2009). Food web models: A plea for groups. Ecology Letters, 12(7), 652–662. 10.1111/j.1461-0248.2009.01321.x 19453619

[ece35641-bib-0004] Banerjee, A. , Scharler, U. M. , Fath, B. D. , & Ray, S. (2017). Temporal variation of keystone species and their impact on system performance in a South African estuarine ecosystem. Ecological Modelling, 363, 207–220. 10.1016/j.ecolmodel.2017.09.009

[ece35641-bib-0005] Barabás, G. , Michalska‐Smith, M. J. , & Allesina, S. (2017). Self‐regulation and the stability of large ecological networks. Nature Ecology & Evolution, 1(12), 1870–1875. 10.1038/s41559-017-0357-6 29062124

[ece35641-bib-0006] Bassett, D. S. , Wymbs, N. F. , Porter, M. A. , Mucha, P. J. , Carlson, J. M. , & Grafton, S. T. (2011). Dynamic reconfiguration of human brain networks during learning. Proceedings of the National Academy of Sciences, 108(18), 7641–7646. 10.1073/pnas.1018985108 PMC3088578 21502525

[ece35641-bib-0007] Bastian, M. , Heymann, S. , & Jacomy, M. (2009). Gephi: An open source software for exploring and manipulating networks. ICWSM, 8, 361–362.

[ece35641-bib-0008] Bond, W. J. (1994). Keystone species In: Schulze E‐D. & Mooney H. A. (Eds.), Biodiversity and ecosystem function (pp. 237–253). Berlin, Heidelberg: Springer.

[ece35641-bib-0009] Bouquet, J.‐M. , Troedsson, C. , Novac, A. , Reeve, M. , Lechtenbörger, A. K. , Massart, W. , … Thompson, E. M. (2018). Increased fitness of a key appendicularian zooplankton species under warmer, acidified seawater conditions. PLoS ONE, 13(1), e0190625 10.1371/journal.pone.0190625 29298334 PMC5752025

[ece35641-bib-0010] Caputi, L. , Carradec, Q. , Eveillard, D. , Kirilovsky, A. , Pelletier, E. , Pierella Karlusich, J. J. , … Iudicone, I. (2019). Community‐level responses to iron availability in open ocean planktonic ecosystems. Global Biogeochemical Cycles, 33(3), 391–419. 10.1029/2018GB006022

[ece35641-bib-0011] Christensen, V. , & Walters, C. J. (2004). Ecopath with Ecosim: Methods, capabilities and limitations. Ecological Modelling, 172(2–4), 109–139. 10.1016/j.ecolmodel.2003.09.003

[ece35641-bib-0012] Cianelli, D. , D'Alelio, D. , Uttieri, M. , Sarno, D. , Zingone, A. , Zambianchi, E. , & Ribera d'Alcalà, M. (2017). Disentangling physical and biological drivers of phytoplankton dynamics in a coastal system. Scientific Reports, 7(1), 15868 10.1038/s41598-017-15880-x 29158517 PMC5696475

[ece35641-bib-0013] Cloern, J. E. , Abreu, P. C. , Carstensen, J. , Chauvaud, L. , Elmgren, R. , Grall, J. , … Yin, K. (2016). Human activities and climate variability drive fast‐paced change across the world's estuarine‐coastal ecosystems. Global Change Biology, 22(2), 513–529. 10.1111/gcb.13059 26242490

[ece35641-bib-0014] Conley, K. R. , Lombard, F. , & Sutherland, K. R. (2018). Mammoth grazers on the ocean's minuteness: A review of selective feeding using mucous meshes. Proceedings of the Royal Society B‐Biological Sciences, 285(1878), 20180056 10.1098/rspb.2018.0056 PMC5966591 29720410

[ece35641-bib-0015] Daily, G. C. , Ehrlich, P. R. , & Haddad, N. M. (1993). Double keystone bird in a keystone species complex. Proceedings of the National Academy of Sciences, 90, 592–594. 10.1073/pnas.90.2.592 PMC45709 11607351

[ece35641-bib-0016] D'Alelio, D. (2017). Biological complexity behind plankton system functioning: Synthesis and perspectives from a marine long term ecological research. Advances in Oceanography and Limnology, 8(2), 187–198. 10.4081/aiol.2017.7194

[ece35641-bib-0017] D'Alelio, D. , Eveillard, D. , Coles, V. J. , Caputi, L. , Ribera d'Alcalà, M. , & Iudicone, D. (2019). Modelling the complexity of plankton communities exploiting omics potential: From present challenges to an integrative pipeline. Current Opinion in Systems Biology, 19, 68–74. 10.1016/j.coisb.2018.10.003

[ece35641-bib-0018] D'Alelio, D. , Libralato, S. , Wyatt, T. , & Ribera d'Alcalà, M. (2016). Ecological‐network models link diversity, structure and function in the plankton food‐web. Scientific Reports, 6, 21806 10.1038/srep21806 26883643 PMC4756299

[ece35641-bib-0019] D'Alelio, D. , Mazzocchi, M. G. , Montresor, M. , Sarno, D. , Zingone, A. , Di Capua, I. , … Ribera d'Alcalà, M. (2015). The green‐blue swing: Plasticity of plankton food‐webs in response to coastal oceanographic dynamics. Marine Ecology, 36(4), 1155–1170. 10.1111/maec.12211

[ece35641-bib-0020] D'Alelio, D. , Montresor, M. , Mazzocchi, M. G. , Margiotta, F. , Sarno, D. , & Ribera d'Alcalà, M. (2016). Plankton food‐webs: To what extent can they be simplified? Advances in Oceanography and Limnology, 7, 67–92. 10.4081/aiol.2016.5646

[ece35641-bib-0021] Dormann, C. F. , Fründ, J. , & Schaefer, H. M. (2017). Identifying causes of patterns in ecological networks: Opportunities and limitations. Annual Review of Ecology Evolution and Systematics, 48, 559–584. 10.1146/annurev-ecolsys-110316-022928

[ece35641-bib-0022] Dunne, J. A. (2006). The structure of food webs In: Pascual M., & Dunne J. A. (Eds.), Ecological networks: Linking structure and dynamics in food webs (pp. 27–86). Santa Fe Institute Studies in the Sciences of Complexity New York: Oxford University Press.

[ece35641-bib-0023] Fortunato, S. , & Hric, D. (2016). Community detection in networks: A user guide. Physics Reports, 659, 1–44. 10.1016/j.physrep.2016.09.002

[ece35641-bib-0024] Fruchterman, T. M. J. , & Reingold, E. M. (1991). Graph drawing by force‐directed placement. Software: Practice and Experience, 21, 1129–1164. 10.1002/spe.4380211102

[ece35641-bib-0025] Grilli, J. , Rogers, T. , & Allesina, S. (2016). Modularity and stability in ecological communities. Nature Communications, 7, 12031 10.1038/ncomms12031 PMC4931019 27337386

[ece35641-bib-0026] Guidi, L. , Chaffron, S. , Bittner, L. , Eveillard, D. , Larhlimi, A. , Roux, S. , … Gorsky, G. (2016). Plankton networks driving carbon export in the oligotrophic ocean. Nature, 532(7600), 465–470. 10.1038/nature16942 26863193 PMC4851848

[ece35641-bib-0027] Hartmann, M. , Grob, C. , Tarran, G. A. , Martin, A. P. , Burkill, P. H. , Scanlan, D. J. , & Zubkov, M. V. (2012). Mixotrophic basis of Atlantic oligotrophic ecosystems. Proceedings of the National Academy of Sciences, 109(15), 5756–5760. 10.1073/pnas.1118179109 PMC3326507 22451938

[ece35641-bib-0028] Ings, T. C. , Montoya, J. M. , Bascompte, J. , Blüthgen, N. , Brown, L. , Dormann, C. F. , … Lauridsen, R. B. (2009). Ecological networks ‐ Beyond food webs. Journal of Animal Ecology, 78(1), 253–269. 10.1111/j.1365-2656.2008.01460.x 19120606

[ece35641-bib-0029] Jordán, F. (2009). Keystone species and food webs. Philosophical Transactions of the Royal Society of London. Series B, Biological Sciences, 364(1524), 1733–1741. 10.1098/rstb.2008.0335 19451124 PMC2685432

[ece35641-bib-0030] Jordán, F. , Liu, W.‐C. , & van Veen, J. F. (2003). Quantifying the importance of species and their interactions in a host‐parasitoid community. Community Ecology, 4(1), 79–88. 10.1556/ComEc.4.2003.1.12

[ece35641-bib-0031] Jordán, F. , & Scheuring, I. (2004). Network ecology: Topological constraints on ecosystem dynamics. Physics of Life Reviews, 1(3), 139–172. 10.1016/j.plrev.2004.08.001

[ece35641-bib-0032] Kemp, J. E. , Evans, D. M. , Augustyn, W. J. , & Ellis, A. G. (2017). Invariant antagonistic network structure despite high spatial and temporal turnover of interactions. Ecography, 40(11), 1315–1324. 10.1111/ecog.02150

[ece35641-bib-0033] Kortsch, S. , Primicerio, R. , Aschan, M. , Lind, S. , Dolgov, A. V. , & Planque, B. (2019). Food‐web structure varies along environmental gradients in a high‐latitude marine ecosystem. Ecography, 42(2), 295–308. 10.1111/ecog.03443

[ece35641-bib-0034] Krause, A. E. , Frank, K. J. , Mason, D. M. , Ulanowicz, R. E. , & Taylor, W. W. (2003). Compartments revealed in food web structure. Nature, 426(6964), 282–285. 10.1038/nature02115 14628050

[ece35641-bib-0035] Lancichinetti, A. , & Fortunato, S. (2009). Community detection algorithms: A comparative analysis. Physical Review E, 80(5), 56117 10.1103/PhysRevE.80.056117 20365053

[ece35641-bib-0056] Le Quéré, C. , Harrison, S. P. , Colin Prentice, I. , Buitenhuis, E. T. , Aumont, O. , Bopp, L. , … Wolf‐Gladrow, D. (2005). Ecosystem dynamics based on plankton functional types for global ocean biogeochemistry models. Global Change Biology, 11(11), 2016–2040. 10.1111/j.1365-2486.2005.1004.x

[ece35641-bib-0036] Libralato, S. , Christensen, V. , & Pauly, D. (2006). A method for identifying keystone species in food web models. Ecological Modelling, 195(3–4), 153–171. 10.1016/j.ecolmodel.2005.11.029

[ece35641-bib-0037] Lima‐Mendez, G. , Faust, K. , Henry, N. , Decelle, J. , Colin, S. , Carcillo, F. , … Raes, J. (2015). Determinants of community structure in the global plankton interactome. Science, 348(6237), 1262073 10.1126/science.1262073 25999517

[ece35641-bib-0038] Link, J. S. , Pranovi, F. , Libralato, S. , Coll, M. , Christensen, V. , Solidoro, C. , & Fulton, E. A. (2015). Emergent properties delineate marine ecosystem perturbation and recovery. Trends in Ecology & Evolution, 30(11), 649–661. 10.1016/j.tree.2015.08.011 26456382

[ece35641-bib-0039] Luczkovich, J. J. , Borgatti, S. P. , Johnson, J. C. , & Everett, M. G. (2003). Defining and measuring trophic role similarity in food webs using regular equivalence. Journal of Theoretical Biology, 220(3), 303–321. 10.1006/jtbi.2003.3147 12468282

[ece35641-bib-0040] Margalef, R. (1991). Networks in ecology In Higashi M. (Ed.), Theoretical studies of ecosystems – the network perspective (pp. 41–57). Cambridge, UK: Cambridge University Press.

[ece35641-bib-0041] May, R. M. (1972). Will a large complex system be stable? Nature, 238(5364), 413–414. 10.1038/238413a0 4559589

[ece35641-bib-0042] Mazzocchi, M. G. , Dubroca, L. , García‐Comas, C. , Capua, I. D. , & Ribera d'Alcalà, M. (2012). Stability and resilience in coastal copepod assemblages: The case of the Mediterranean long‐term ecological research at Station MC (LTER‐MC). Progress in Oceanography, 97, 135–151. 10.1016/j.pocean.2011.11.003

[ece35641-bib-0043] Montoya, D. , Yallop, M. L. , & Memmott, J. (2015). Functional group diversity increases with modularity in complex food webs. Nature Communications, 6, 7379 10.1038/ncomms8379 PMC4490355 26059871

[ece35641-bib-0044] Montoya, J. M. , & Solé, R. V. (2002). Small world patterns in food webs. Journal of Theoretical Biology, 214(3), 405–412. 10.1006/jtbi.2001.2460 11846598

[ece35641-bib-0045] Morris, R. J. , Lewis, O. T. , & Godfray, H. C. J. (2004). Experimental evidence for apparent competition in a tropical forest food web. Nature, 428(6980), 310–313. 10.1038/nature02394 15029194

[ece35641-bib-0046] Moustaka‐Gouni, M. , Kormas, K. A. , Scotti, M. , Vardaka, E. , & Sommer, U. (2016). Warming and acidification effects on planktonic heterotrophic pico‐and nanoflagellates in a mesocosm experiment. Protist, 167(4), 389–410. 10.1016/j.protis.2016.06.004 27472657

[ece35641-bib-0047] Muller, C. B. , Adriaanse, I. C. T. , Belshaw, R. , & Godfray, H. C. J. (1999). The structure of an aphid‐parasitoid community. Journal of Animal Ecology, 68(2), 346–370. 10.1046/j.1365-2656.1999.00288.x

[ece35641-bib-0048] Müller, C. B. , & Godfray, H. C. J. (1999). Indirect interactions in aphid‐parasitoid communities. Population Ecology, 41(1), 93–106. 10.1007/PL00011986

[ece35641-bib-0049] Ortiz, M. , Levins, R. , Campos, L. , Berrios, F. , Campos, F. , Jordán, F. , … Rodriguez, F. (2013). Identifying keystone trophic groups in benthic ecosystems: Implications for fisheries management. Ecological Indicators, 25, 133–140. 10.1016/j.ecolind.2012.08.020

[ece35641-bib-0050] Paffenhöfer, G.‐A. , Sherr, B. F. , & Sherr, E. B. (2007). From small scales to the big picture: Persistence mechanisms of planktonic grazers in the oligotrophic ocean. Marine Ecology, 28(2), 243–253. 10.1111/j.1439-0485.2007.00162.x

[ece35641-bib-0051] Peacor, S. D. , Riolo, R. L. , & Pascual, M. (2006). Phenotypic plasticity and species coexistence: Modeling food webs as complex adaptive systems In: Pascual M., & Dunne J. A. (Eds.), Ecological networks: Linking structure and dynamics in food webs (pp. 245–270). Santa Fe Institute Studies in the Sciences of Complexity. New York: Oxford University Press.

[ece35641-bib-0052] Peralta, G. , Frost, C. M. , Didham, R. K. , Rand, T. A. , & Tylianakis, J. M. (2017). Non‐random food‐web assembly at habitat edges increases connectivity and functional redundancy. Ecology, 98(4), 995–1005. 10.1002/ecy.1656 27859031

[ece35641-bib-0053] Pilosof, S. , Porter, M. A. , Pascual, M. , & Kéfi, S. (2017). The multilayer nature of ecological networks. Nature Ecology & Evolution, 1(4), 0101 10.1038/s41559-017-0101 28812678

[ece35641-bib-0054] Poisot, T. , Canard, E. , Mouillot, D. , Mouquet, N. , & Gravel, D. (2012). The dissimilarity of species interaction networks. Ecology Letters, 15(12), 1353–1361. 10.1111/ele.12002 22994257

[ece35641-bib-0055] Poisot, T. , Stouffer, D. B. , & Gravel, D. (2015). Beyond species: Why ecological interaction networks vary through space and time. Oikos, 124(3), 243–251. 10.1111/oik.01719

[ece35641-bib-0057] R Development Core Team (2008). R: A language and environment for statistical computing.

[ece35641-bib-0058] Rezende, E. L. , Albert, E. M. , Fortuna, M. A. , & Bascompte, J. (2009). Compartments in a marine food web associated with phylogeny, body mass, and habitat structure. Ecology Letters, 12(8), 779–788. 10.1111/j.1461-0248.2009.01327.x 19490028

[ece35641-bib-0059] Ribera d'Alcalà, M. , Conversano, F. , Corato, F. , Licandro, P. , Mangoni, O. , Marino, D. , … Zingone, A. (2004). Seasonal patterns in plankton communities in a pluriannual time series at a coastal Mediterranean site (Gulf of Naples): An attempt to discern recurrences and trends. Scientia Marina, 68(S1), 65–83. 10.3989/scimar.2004.68s165

[ece35641-bib-0060] Rooney, N. , McCann, K. S. , & Moore, J. C. (2008). A landscape theory for food web architecture. Ecology Letters, 11(8), 867–881. 10.1111/j.1461-0248.2008.01193.x 18445027

[ece35641-bib-0061] Rosvall, M. , Axelsson, D. , & Bergstrom, C. T. (2009). The map equation. The European Physical Journal Special Topics, 178(1), 13–23. 10.1140/epjst/e2010-01179-1

[ece35641-bib-0062] Rosvall, M. , & Bergstrom, C. T. (2008). Maps of random walks on complex networks reveal community structure. Proceedings of the National Academy of Sciences, 105(4), 1118–1123. 10.1073/pnas.0706851105 PMC2234100 18216267

[ece35641-bib-0063] Rott, A. S. , & Godfray, H. C. J. (2000). The structure of a leafminer‐parasitoid community. Journal of Animal Ecology, 69(2), 274–289. 10.1046/j.1365-2656.2000.00390.x

[ece35641-bib-0064] Schmitz, O. J. , Krivan, V. , & Ovadia, O. (2004). Trophic cascades: The primacy of trait‐mediated indirect interactions. Ecology Letters, 7(2), 153–163. 10.1111/j.1461-0248.2003.00560.x

[ece35641-bib-0065] Scotti, M. , Podani, J. , & Jordán, F. (2007). Weighting, scale dependence and indirect effects in ecological networks: A comparative study. Ecological Complexity, 4(3), 148–159. 10.1016/j.ecocom.2007.05.002

[ece35641-bib-0066] Shannon, L. J. , & Cury, P. M. (2004). Indicators quantifying small pelagic fish interactions: Application using a trophic model of the southern Benguela ecosystem. Ecological Indicators, 3(4), 305–321. 10.1016/j.ecolind.2003.11.008

[ece35641-bib-0067] Stibor, H. , Vadstein, O. , Diehl, S. , Gelzleichter, A. , Hansen, T. , Hantzsche, F. , … Olsen, Y. (2004). Copepods act as a switch between alternative trophic cascades in marine pelagic food webs. Ecology Letters, 7(4), 321–328. 10.1111/j.1461-0248.2004.00580.x

[ece35641-bib-0068] Stoecker, D. K. , Hansen, P. J. , Caron, D. A. , & Mitra, A. (2017). Mixotrophy in the marine plankton. Annual Review of Marine Science, 9, 311–335. 10.1146/annurev-marine-010816-060617 27483121

[ece35641-bib-0069] Stouffer, D. B. , & Bascompte, J. (2011). Compartmentalization increases food‐web persistence. Proceedings of the National Academy of Sciences, 108(9), 3648–3652. 10.1073/pnas.1014353108 PMC3048152 21307311

[ece35641-bib-0070] Thébault, E. , & Fontaine, C. (2010). Stability of ecological communities and the architecture of mutualistic and trophic networks. Science, 3299(5993), 853–857. 10.1126/science.1188321 20705861

[ece35641-bib-0071] Thompson, R. M. , Brose, U. , Dunne, J. A. , Hall, R. O. , Hladyz, S. , Kitching, R. L. , … Tylianakis, J. M. (2012). Food webs: Reconciling the structure and function of biodiversity. Trends in Ecology & Evolution, 27(12), 689–697. 10.1016/j.tree.2012.08.005 22959162

[ece35641-bib-0072] Trøjelsgaard, K. , & Olesen, J. M. (2016). Ecological networks in motion: Micro‐ and macroscopic variability across scales. Functional Ecology, 30(12), 1926–1935. 10.1111/1365-2435.12710

[ece35641-bib-0073] Tylianakis, J. M. , & Morris, R. J. (2017). Ecological networks across environmental gradients. Annual Review of Ecology Evolution and Systematics, 48, 25–48. 10.1146/annurev-ecolsys-110316-022821

[ece35641-bib-0074] Ursella, L. , Cardin, V. , Batistić, M. , Garić, R. , & Gačić, M. (2018). Evidence of zooplankton vertical migration from continuous Southern Adriatic buoy current‐meter records. Progress in Oceanography, 167, 78–96. 10.1016/j.pocean.2018.07.004

[ece35641-bib-0075] Valentini, R. , & Jordán, F. (2010). CoSBiLab Graph: The network analysis module of CoSBiLab. Environmental Modelling and Software, 25(7), 886–888. 10.1016/j.envsoft.2010.02.001

[ece35641-bib-0076] Wear, E. K. , Carlson, C. A. , James, A. K. , Brzezinski, M. A. , Windecker, L. A. , & Nelson, C. E. (2015). Synchronous shifts in dissolved organic carbon bioavailability and bacterial community responses over the course of an upwelling‐driven phytoplankton bloom. Limnology and Oceanography, 60(2), 657–677. 10.1002/lno.10042

[ece35641-bib-0077] Wiese, R. , Eiglsperger, M. , & Kaufmann, M. (2004). yFiles — Visualization and automatic layout of graphs In: Jünger M. & Mutzel P. (Eds.), Graph Drawing Software. Mathematics and Visualization (pp. 173–191). Berlin, Heidelberg: Springer.

[ece35641-bib-0078] Winder, M. , Bouquet, J.‐M. , Rafael Bermúdez, J. , Berger, S. A. , Hansen, T. , Brandes, J. , … Thompson, E. M. (2017). Increased appendicularian zooplankton alter carbon cycling under warmer more acidified ocean conditions. Limnology and Oceanography, 62(4), 1541–1551. 10.1002/lno.10516

