# Supplementary material for: Rewiring and indirect effects underpin modularity reshuffling in a marine food web under environmental shifts
Source: Ecol Evol. 2019 Sep 30;9(20):11631–46. doi: 10.1002/ece3.5641 (PMC6822054; doi:10.1002/ece3.5641)
Supplement: Supplementary file 3 [file ECE3-9-11631-s003.docx]

**Supplementary methods**

**Rewiring and indirect effects underpin modularity reshuffling in a marine food web under environmental shifts**

Domenico D’Alelio^1,*^, Bruno Hay Mele^1^, Simone Libralato^2^, Maurizio Ribera d’Alcalà^1,2^, Ferenc Jordán^1,3^

^1^ Stazione Zoologica Anton Dohrn, Napoli (Italy)

^2^ National Institute of Oceanography and Applied Geophysics – OGS, Trieste (Italy)

^3^ Balaton Limnological Institute, MTA Centre for Ecological Research, Tihany (Hungary)

**Data S3.**

***INE estimation***

We calculated the weighted net indirect effect (*INE*) on the base of the net effect between two nodes in the weighted network. First, we define the net effect of node i on node j (q_ij_) as the difference between positive effects, quantified by the fraction of the prey i in the diet of the predator j (d_ji_), and negative effects, evaluated through the fraction of total consumption of i used by predator j (f_ij_). The coefficients of the resulting matrix of the net impacts (Q) will then have the form

$$q_{i,j}=d_{j,i}-f_{i,j}=\frac{\mu_{i,j}}{\sum_{k=1,N} \mu_{k,j}}-\frac{\mu_{i,j}}{\sum_{m=1,N} \mu_{i,m}}$$

Where $\mu_{i,j}$is the weighted link between each prey i and predator j. The product of all the net impacts q_ij_ for all the possible pathways that link the functional groups i and j in the trophic web allows calculating the mixed trophic impact, that cumulates direct and indirect effects (Ulanowicz & Puccia 1990). Considering the net effects as multiplicative along the paths, the impact from node i to node j linked by the path including, for example, nodes w, z, v is

$$\left. p_{i,j} \right|_{w,z,v}=q_{i,w}\cdot q_{w,z}\cdot q_{z,v}\cdot q_{v,j}$$

with the total effect of a node on another node considered as the sum of the effects along all the possible pathways in the web (briefly indicated as h). Thus, the total mixed trophic impact is

$$m_{i,j}=\sum_{h=1}^{H} \left. p_{i,j} \right|_{h}$$

where H is the total number of possible pathways (a very large number). The m_ij_ quantify the direct and indirect impacts that each (impacting) functional group i has on any (impacted) functional group j of the food web (Ulanowicz & Puccia 1990). Positive/negative values of m_ij_ indicate the increase/decrease of the biomass of the group j due to a slight increase/decrease of the biomass of the impacting group i (Libralato *et al.* 2006). INE was calculated as the sum of all impacts involving a group disregarding their sign, i.e. considering their absolute value:

$$INE_{i}=\sum_{j\neq i}^{N} \left| m_{ij} \right|$$

***WI and WO estimation***

In an unweighted network, we define *a_n,ij_* as the effect of *j* on *i* when *j* can reach *i* in *n* steps.

The simplest mode of calculating *a_n,ij_* is when *n* = 1 (*i.e.* the effect of *j* on *i* in 1 step): *a*_1_,*_ij_* = 1/*D_i_*, where *D_i_* is the degree of node *i* (*i.e.* the number of its direct neighbours including both prey or predatory species). We again assumed that indirect chain effects were multiplicative and additive. The effect of *j* on *i* in 2 steps (through an intermediate *k* node) equals *a*_1_,*_kj_* · *a*_1_,*_ik_* (multiplicative). If there are two such 2-step pathways from *j* to *i* (one is through *k* and the other through *h*), the effect of *j* on *i* equals *a*_2_,*_ij_*= *a*_1_,*_kj_* · *a*_1_,*_ik_*+ *a*_1_,*_hj_* · *a*_1_,*_ih_* (additive).

After considering effects of step *n*, we defined the effect received by species *i* from all species in the same network as:

$$\psi_{n,i}=\sum_{j=1}^{N} a_{n,ij}$$

which is equal to 1 (*i.e.* each species is affected by the same unit effect). Furthermore, we defined the *n*-step effect originated from a species *i* as:

$$\sigma_{n,i}=\sum_{j=1}^{N} a_{n,ji}$$

which may vary among different species (*i.e.* effects originated from different species can be different). Here, we define the topological importance of species *i* when effects up to the *n-th* step are considered as:

$$TI_{i}^{n}=\frac{\sum_{m=1}^{n} \sigma_{m,i}}{n}=\frac{\sum_{m=1}^{n} \sum_{j=1}^{N} a_{m,ji}}{n}$$

which is merely the sum of effects originated from species *i* up to *n* steps (one plus two plus three…up to *n*) divided by *n*. For a weighted network, all effects were defined in the same way as above, but the 1-step effect was defined as:

$$a_{1,ij}=\frac{\varepsilon_{ij}}{\mu_{i}}$$

where *μ_i_* is the summed weights of links connected to *i* and *ε_ij_* is the strength of the link connecting *i* to *j*. The weighted approach of calculating 2-step effects (*i.e.* *a*_2_,*_ij_*) was initially developed for assessing apparent competition in host-parasitoid communities (Müller & Godfray 1999). Furthermore, we defined *WI_i_^n^* as the topological importance of species *i* for networks with weighted links when effects up to *n* steps are considered:

$$WI_{i}^{n}=\frac{\sum_{m=1}^{n} \sigma_{m,i}}{n}=\frac{\sum_{m=1}^{n} \sum_{j=1}^{N} a_{m,ji}}{n}$$

For the calculations of *WI* (see also (Jordán *et al.* 2003)), we considered n = 3 (maximum three steps for indirect effects), and we used the CosbiLab Graph software for the calculations (Valentini & Jordán 2010).

The vector of *a_n,ij_*-values for species *j* has been defined as its ‘trophic field’ (Jordán 2001). For long indirect effects, every species connects to every other. It is reasonable to define a *t* threshold of *a_n,ij_*-values separating stronger interactive partners from the weaker ones. Given a maximum length of indirect effects (*n*) and a threshold for interaction strength (*t*), every node may be characterised by its effective trophic field. Since the sets of strong interactors of two nodes may overlap, it is important to quantify the positional uniqueness of graph nodes.

The number of strong interactors appearing in the effective trophic fields of nodes *i* and *j* are expressed as a function of *n* and *t* by the ‘trophic field overlap’ (*TO^n,t^_ij_*) between nodes *i* and *j*. The sum of all *TO*-values between species *i* and others (Σ*TO^n,t^_ij_* summed over all *j*) provides the summed trophic field overlap of species *i* (*TO^n,t^_i_*). We used its normalised value, dividing it by the maximum possible value of 2*N*, where *N* is the number of nodes in the network. *TO* quantifies the redundancy in the network and low *TO* values mark nodes in unique network positions (poor overlaps).

Based on the interaction matrix given for a weighted network, we determined the weighted overlap (*WO*) for each species. We calculated the *WO^3,1000000^*-values for each species, so the threshold chosen was *t* = 10^6^, based on the analysis of the interaction matrix. Doing so, *l^s^* = 1964 out of *l^max^* = *n*^2^ = 62^2^ = 3844 effects have been considered ‘strong’; if the threshold was lower, too many interactions would be considered as strong, while a higher threshold provides only a few overlaps (*l^s^* = 962 for *t* = 10^7^, *l^s^* = 397 for *t* = 10^8^, *l^s^* = 138 for *t* = 10^9^, *l^s^* = 38 for *t* = 10^10^). The *WO* index cannot provide meaningful information in either case.

References

Jordán, F. (2001). Trophic fields. *Community Ecol.*, 2, 181–185.

Jordán, F., Liu, W.-C. & van Veen, J.F. (2003). Quantifying the importance of species and their interactions in a host-parasitoid community. *Community Ecol.*, 4, 79–88.

Libralato, S., Christensen, V. & Pauly, D. (2006). A method for identifying keystone species in food web models. *Ecol. Modell.*, 195, 153–171.

Müller, C.B. & Godfray, H.C.J. (1999). Indirect interactions in aphid--parasitoid communities. *Popul. Ecol. (Kyoto).*, 41, 93–106.

Ulanowicz, R.E. & Puccia, C.J. (1990). Mixed trophic impacts in ecosystems. *Coenoses*, 5, 7–16.

Valentini, R. & Jordán, F. (2010). CoSBiLab Graph: the network analysis module of CoSBiLab. *Environ. Model. Softw.*, 25, 886–888.
